# Exploring the relationship between post-contrast acute kidney injury and different baseline creatinine standards: A retrospective cohort study

**DOI:** 10.3389/fendo.2022.1042312

**Published:** 2023-01-12

**Authors:** Jixiang Ying, Junren Wang, Zhiye Ying, Xingwu Ran, Xiaoxi Zeng, Dawei Chen, Yun Gao, Li Zhong

**Affiliations:** ^1^ Department of Endocrinology and Metabolism, Diabetic Foot Care Center, West China Hospital, Sichuan University, Chengdu, China; ^2^ Department of Endocrinology and Metabolism, West China Longquan Hospital Sichuan University, and The First People’s Hospital of Longquanyi District, Chengdu, China; ^3^ West China Biomedical Big Data Center, West China Hospital, Sichuan University, Chengdu, China; ^4^ Med-X Center for Informatics, Sichuan University, Chengdu, China; ^5^ West China Biomedical Big Data Center, Division of Nephrology, Kidney Research Institute, West China Hospital, Sichuan University, Chengdu, China

**Keywords:** diabetes mellitus, epidemiology, digital subtraction angiography, enhanced CT, post-contrast acute kidney injury

## Abstract

**Objective:**

According to previous studies, the incidence of post-contrast acute kidney injury(PC-AKI) in diabetic is far higher than that in the general population. Therefore, we explored the relationship between the incidence of PC-AKI and different baseline serum creatinine (SCr) levels, and determined the relationship between PC-AKI and different types of contrast media (CMs), different doses of CM, and different examination methods in this specific population.

**Materials and methods:**

Patients with diabetes in whom CM was used between 2010 and 2020 at our institution were included. Participants were identified according to the following three schemes: Scheme 1 (n=5911), SCr was detected before and within 72 h after using CM; Scheme 2 (n=2385), SCr was detected within 24 h before and within 24–72 h after using CM; and Scheme 3 (n=81), SCr was detected within 24 h before and within 0–24, 24–48, and 48–72 h after using CM. The incidence of PC-AKI with different types of CM, incidence of PC-AKI on digital subtraction angiography (DSA) and enhanced computed tomography (CT), proportion of PC-AKI with different doses of CM, and baseline SCr at different stages of PC-AKI were compared. Multivariate logistic regression analysis was used to explore risk factors for PC-AKI.

**Results:**

A total of 29,081 patients were included in this study. The incidence of PC-AKI in Scheme 3 (22.22%) was higher than those in Schemes 1 (6.19%) and 2 (7.71%). The incidence of PC-AKI on DSA was higher than that on enhanced CT (8.30% vs. 5.80%; *P*<0.05). The incidence of PC-AKI in the increased-dose CM group was higher than that in the non-increased-dose CM group (7.9% vs. 5.7%; *P*<0.01). Moreover, there were differences in baseline SCr values at different stages of PC-AKI (*P*<0.01). Multivariate logistic regression analysis showed that hypertension, chronic kidney disease, heart failure, peripheral vascular disease, metformin, diuretics, and CM dose were risk factors for PC-AKI.

**Conclusion:**

The incidence of PC-AKI increased significantly with increasing time requirement and frequency of SCr detection. Moreover, before using CM, we should control the blood pressure and heart failure, stop using metformin and diuretics, and use CMs at the minimum dose to avoid PC-AKI.

## 1 Introduction

The application of contrast media (CMs) for diagnostic imaging is an important part of the clinical diagnosis and treatment processes. With the widespread application of imaging and interventional techniques in the clinical diagnosis and treatment processes, post-contrast acute kidney injury (PC-AKI) has become the third leading cause of incomplete iatrogenic renal function (1–4). The incidence of PC-AKI in the general population is approximately 2.7% (5), while that in patients with diabetes mellitus (DM), which is a known high-risk group for PC-AKI, is 5.7% to 29.4% (6). Furthermore, the prevalence of diabetes has been gradually increasing. According to the IDF Diabetes Atlas 10th edition (7), the global prevalence of diabetes among people aged 20–79 in 2021 is estimated to be 9.8% (536.6 million people). Approximately 0.4%–5.9% of PC-AKI patients require dialysis treatment, whereas in approximately 0.4%–3.1% PC-AKI patients, renal function cannot be restored for life (2). PC-AKI can lead to prolonged hospitalization, increased costs of diagnosis and treatment, and higher risk of death (8).

In 2020, the American College of Radiology and National Kidney Foundation jointly issued a consensus on the use of intravenous iodine CMs in patients with kidney disease (9), which was consistent with the guidelines of the European Society of Urogenital Radiology Contrast Medium Safety Committee in 2018 (10). PC-AKI was defined as acute renal impairment after the use of CM, occurring within 48–72 hours, and a serum creatinine (SCr) increase by 0.3 mg/dL (26.5 µmol/L) or ≥1.5 times the baseline level. This requires that SCr be detected immediately before using CM; however, in practice, this is challenging. Consequently, it is possible that the detection time of baseline SCr is relatively delayed from the application of CM and that the peak of PC-AKI occurs within 48–72 hours (11). If SCr is not detected within 24 h before CM use or is only detected within 24 h after application, it is difficult to rule out whether acute renal impairment has occurred before CM use. Therefore, it is necessary to specify for how long the SCr detection value can be used as the baseline value before using the CM.

This study aimed to calculate the incidence of PC-AKI in patients with DM using different SCr detection schemes, explore the relationship between the incidence of PC-AKI and different baseline SCr standards, and investigate the epidemiological characteristics of PC-AKI in this specific population.

## 2 Materials and methods

### 2.1 Study participants

Patients with DM who were hospitalized in our institution between 2010 and 2020 and in whom iodine CM was used were included in this study. The inclusion criteria were as follows: (1) SCr was detected at least twice before and within 72 h after using CM; and (2) patients’ first use of CM in this hospitalization was included if CM was used multiple times. The exclusion criteria were as follows: (1) patients with stage 5 chronic kidney disease (CKD) and (2) patients who had undergone dialysis treatment before using CM.

### 2.2 Research design

This retrospective study was registered in the Chinese Clinical Trial Registry (registration number: ChiCTR2100041717). The primary outcome was PC-AKI incidence. Three SCr detection schemes were used to determine the incidence of PC-AKI. In Scheme 1, SCr was detected before and within 72 h after using CM; in Scheme 2, SCr was detected within 24 h before and within 24–72 h after using CM; and in Scheme 3, SCr was detected within 24 h before and within 0–24, 24–48 and 48–72 h after using CM.

The secondary outcomes included the following: 1) the proportion of PC-AKI caused by different types of CM and different doses of CM; 2) the incidence of PC-AKI examined by digital subtraction angiography (DSA) and enhanced computed tomography (CT); 3) the baseline SCr value of PC-AKI at different times and at different stages of PC-AKI; and 4) risk factors for PC-AKI.

According to the consensus (8) PC-AKI staging standard, the following stages were defined: stage 1: 1.5–1.9 times baseline SCr or increase in SCr ≥0.3 mg/dL (≥26.5 µmol/L); stage 2: 2.0–2.9 times baseline SCr; stage 3: 3.0 times baseline SCr or increase in SCr ≥4.0 mg/dL (≥353.6 µmol/L), or initiation of kidney replacement therapy.

### 2.3 Data sources

All the data used in this study were obtained from the electronic health record system of our institution.

### 2.4 Statistical methods

Normally distributed continuous data are expressed as mean ± standard deviation, and pairwise comparisons between the sample groups were performed using the t-test. Non-normally distributed continuous data are expressed as the median (interquartile range), and pairwise comparisons between the sample groups were performed using the rank-sum test. The categorized data are expressed as numbers (rates), and comparisons between the groups were performed using the chi-square test or Fisher’s exact test. Multivariate logistic regression analysis was conducted to explore the risk factors of PC-AKI. *P*-values were adjusted for multiple pairwise comparisons using the Bonferroni method, and *P* ≤ 0.05 indicated a statistically significant difference. SPSS 22.0 software was used for data analysis, and the results were visualized using Excel.

## 3 Results

**Figure 1:**
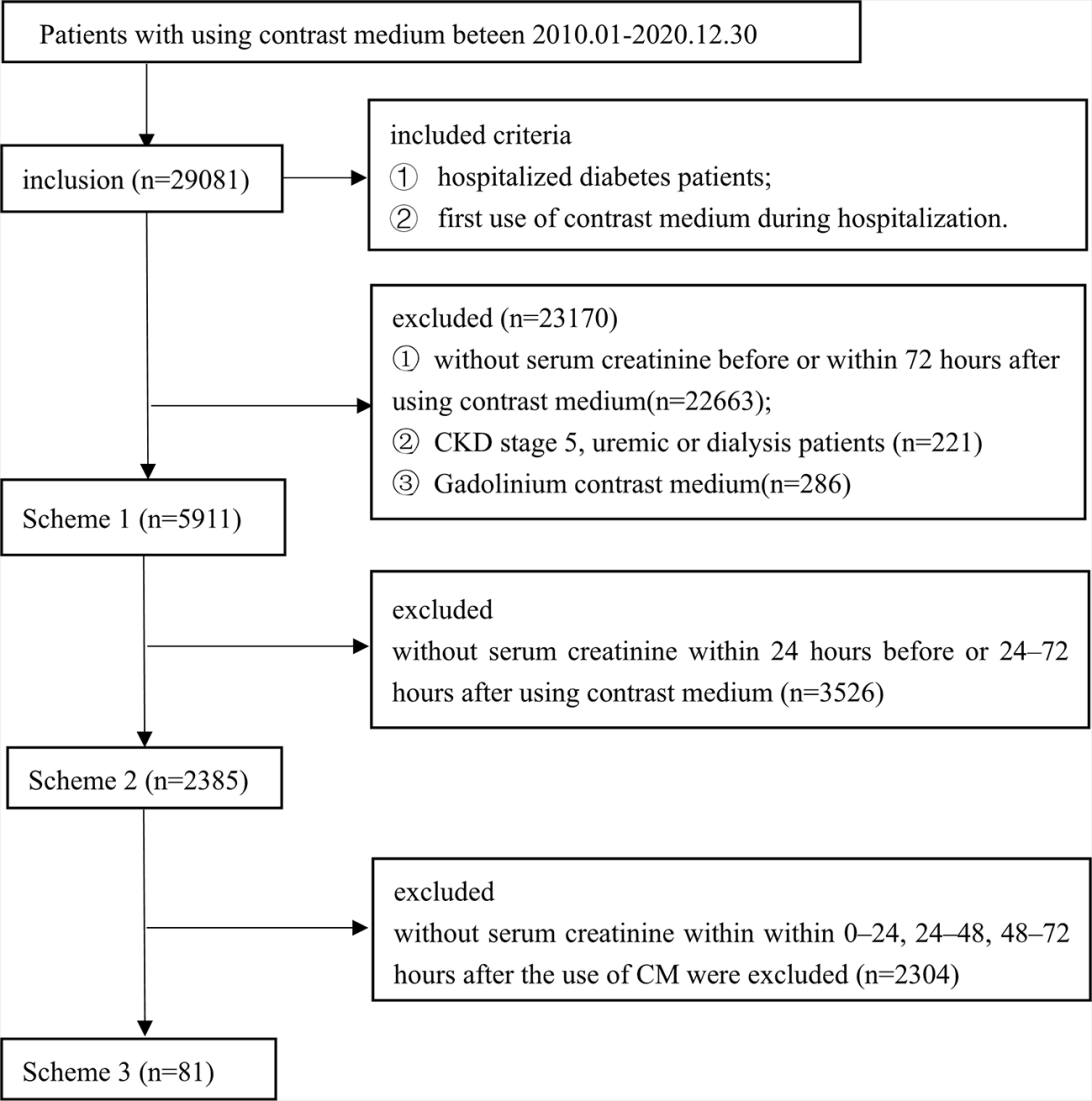
Flow diagram of included studies. Three different serum creatinine detection standard schemes: Scheme 1 (n=5911), SCr was detected before and within 72 h after using contrast medium; Scheme 2 (n=2385), SC was detected within 24 h before and within 24-72 h after using contrast medium; and Scheme 3 (n=81), SCr was detected within 24 h before and within 0-24, 24-48, and 48-72 h after using contrast medium.

In this study, data from 29,081 patients’ first use of CM during hospitalization were included. However, 22,663 were excluded because they did not have data on SCr levels before or within 72 h after using CM. Only 6,418 patients had data on SCr detection values before and within 72 h after CM application. After excluding patients with stage 5 CKD, or dialysis before using CM, 5,911 patients were finally included in Scheme 1. A flowchart of the patient screening process is shown in **Figure 1**. There were 366 patients (6.19%) with PC-AKI, of which 249 (68.0%) were male and 117 (32.0%) were female, and the difference was not statistically significant (*P*=0.288).

### 3.1 Daily SCr detection rate and PC-AKI incidence within 7 days after administration of CM

In this study, the diagnostic criteria for PC-AKI occurring 4-7 days after using CM was defined as the same as that for PC-AKI occurring within 72 hours.The SCr detection rate in the first three days after the use of iodine CM was relatively high (26.3–30.8%), which was significantly different from that in the fourth to seventh days (11.3–20.1%) (*P*<0.05). However, there was no statistically significant difference in the new incidence of PC-AKI in daily detection (*P*>0.05) (**Tables 1**, **2**).

**Table 1:** Comparison of daily serum creatinine detection rate within 7 days after using contrast medium.

Time	Detected amount	Pending PC-AKI	%	*χ* ^2^	*p-*value
Day 1	2182	5911	27.0%_a_	1875.636	<0.01
Day 2	2032	5682	26.3%_a_		
Day 3	2463	5545	30.8%_b_		
Day 4	703	5509	11.3%_c_		
Day 5	854	5462	13.5%_d_		
Day 6	817	5425	12.4%_c,d_		
Day 7	763	5394	20.1%_c,d_		

Different subscript letters in the PC-AKI group indicate significant differences between the two groups (P<0.05). PC-AKI, post-contrast acute kidney injury.

**Table 2:** Comparison of new incidence of PC-AKI detected daily after using contrast medium.

Time	Detected amount	New PC-AKI	%	*χ* ^2^	*p-*value
Day 1	2182	117	5.1%	3.581	0.733
Day 2	2032	112	5.2%		
Day 3	2463	137	5.3%		
Day 4	703	36	4.9%		
Day 5	854	47	5.2%		
Day 6	817	37	4.3%		
Day 7	763	31	3.9%		

PC-AKI, post-contrast acute kidney injury.

### 3.2 Incidence of PC-AKI with different iodine CMs

This study included seven types of iodine CMs, and there was no statistically significant difference in the incidence of PC-AKI caused by various iodine CMs (*P*>0.05) (**Table 3**).

**Table 3:** Comparison of the incidence of PC-AKI caused by different iodine contrast mediums.

Contrast medium	PC-AKI	%	non-PC-AKI	%	*χ* ^2^	*p-*value
Iopamidol	87	7.7%	1050	92.3%	11.919	0.064
Iohexol	54	6.5%	773	93.5%		
Iopromide	31	5.1%	577	94.9%		
Iobitridol	31	5.9%	491	94.1%		
Ioversol	19	5.8%	307	94.2%		
Iodixanol	129	5.7%	2149	94.3%		
Iomeprol	37	9.0%	373	91.0%		

PC-AKI, post-contrast acute kidney injury.

### 3.3 Incidence of PC-AKI according to different examination methods

**Figure 2:**
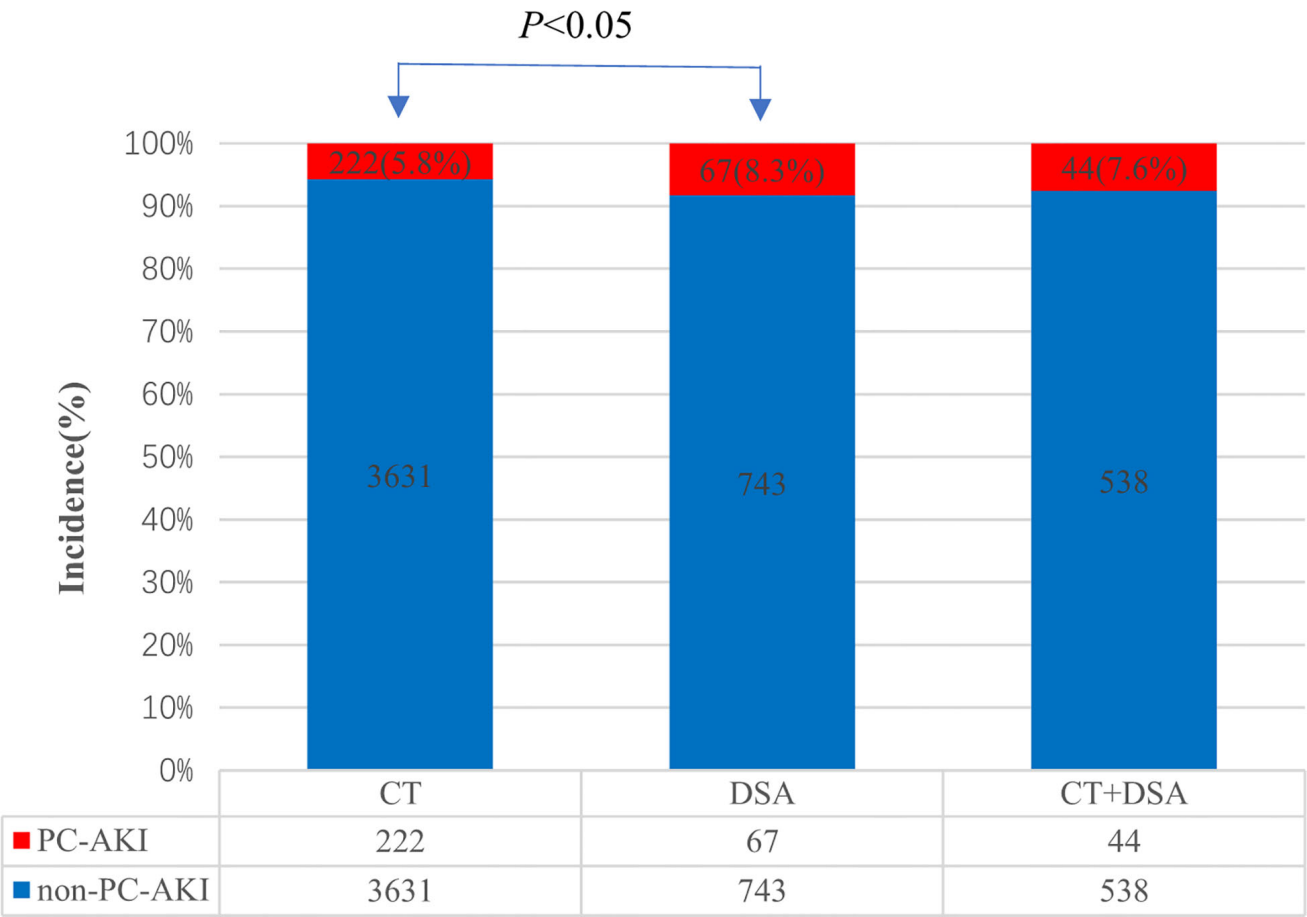
Incidence of PC-AKI in different examination methods.

DSA and enhanced CT were performed using iodine CM. In Scheme 1, DSA was used in 810 cases, of which 67 (8.3%) developed PC-AKI. Meanwhile, enhanced CT examination was used in 3,853 cases, of which 222 (5.8%) developed PC-AKI. Moreover, the two examination methods were used concurrently in 582 cases, of which 44 (7.6%) developed PC-AKI. The results showed that DSA had a significantly higher incidence of PC-AKI than did enhanced CT (*P*<0.05). For the concurrent application of two examinations, the incidence of PC-AKI was lower than that of DSA and higher than that of enhanced CT, but the difference was not statistically significant (*P*>0.05) (**Figure 2**).

### 3.4 Relationship between the dose of iodine CM and PC-AKI

This study defined an iodine CM dose of ≥100 mL as an increase in the CM dose. A total of 1,283 cases with increased CM doses were recorded, of which 101 (7.9%) cases of PC-AKI occurred. Meanwhile, 4,629 cases showed no increase in CM dose, among which PC-AKI occurred in 265 (5.7%) cases. The incidence of PC-AKI in the two groups was significantly different (*P*<0.01).

### 3.5 Comparison of PC-AKI incidence in the three schemes

**Figure 3:**
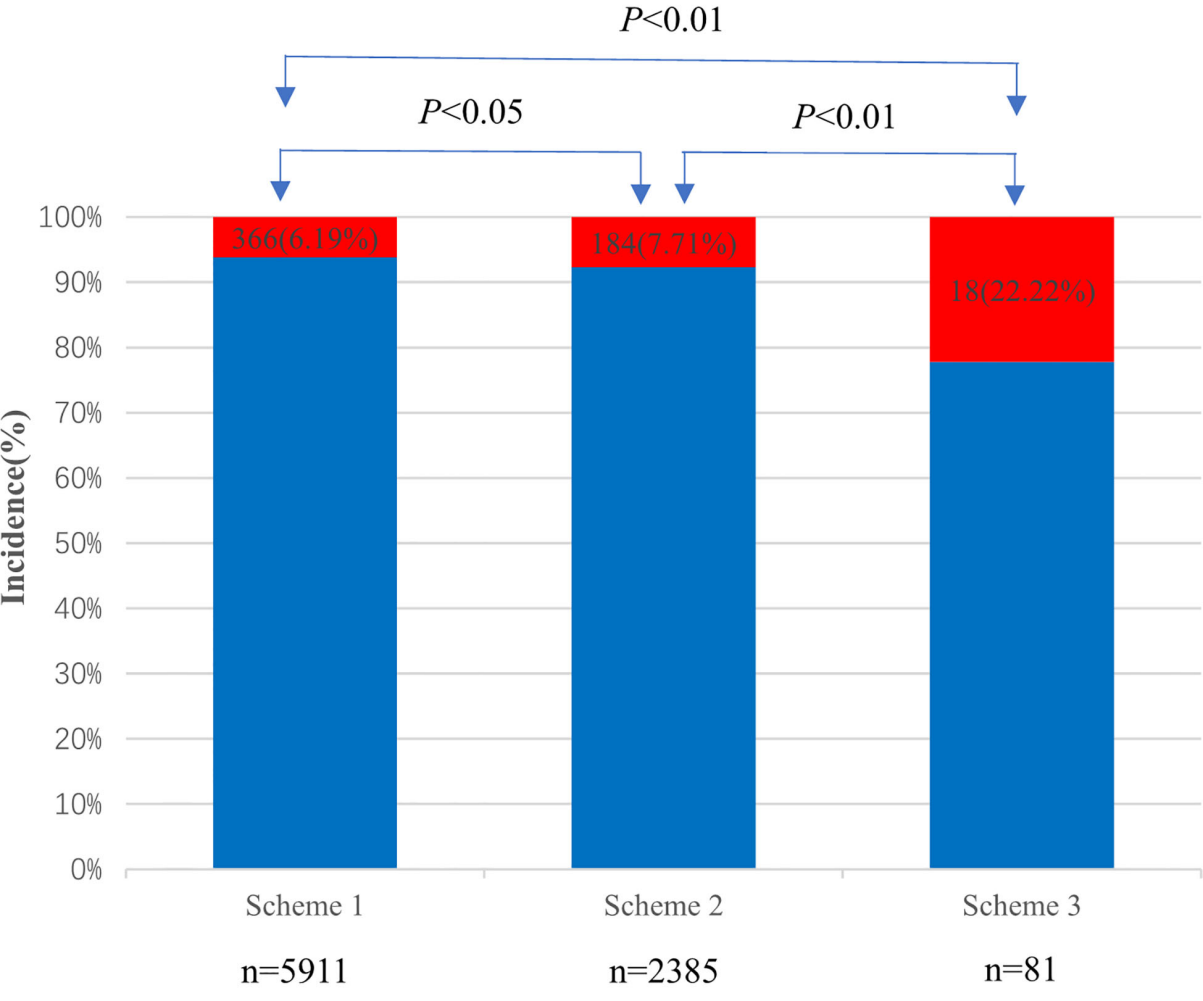
Incidence of PC-AKI in three schemes.

A total of 2,385 patients were included in Scheme 2, of which 184 (7.71%) developed PC-AKI within 72 h. Meanwhile, 81 cases were included in Scheme 3, of which 18 (22.22%) developed PC-AKI within 72 h. The incidence of PC-AKI among the three groups was statistically significant (*P*<0.05) (**Figure 3**).

### 3.6 Comparison of baseline creatinine values in the PC-AKI, non-PC-AKI, and PC-AKI groups at different stages

**Figure 4:**
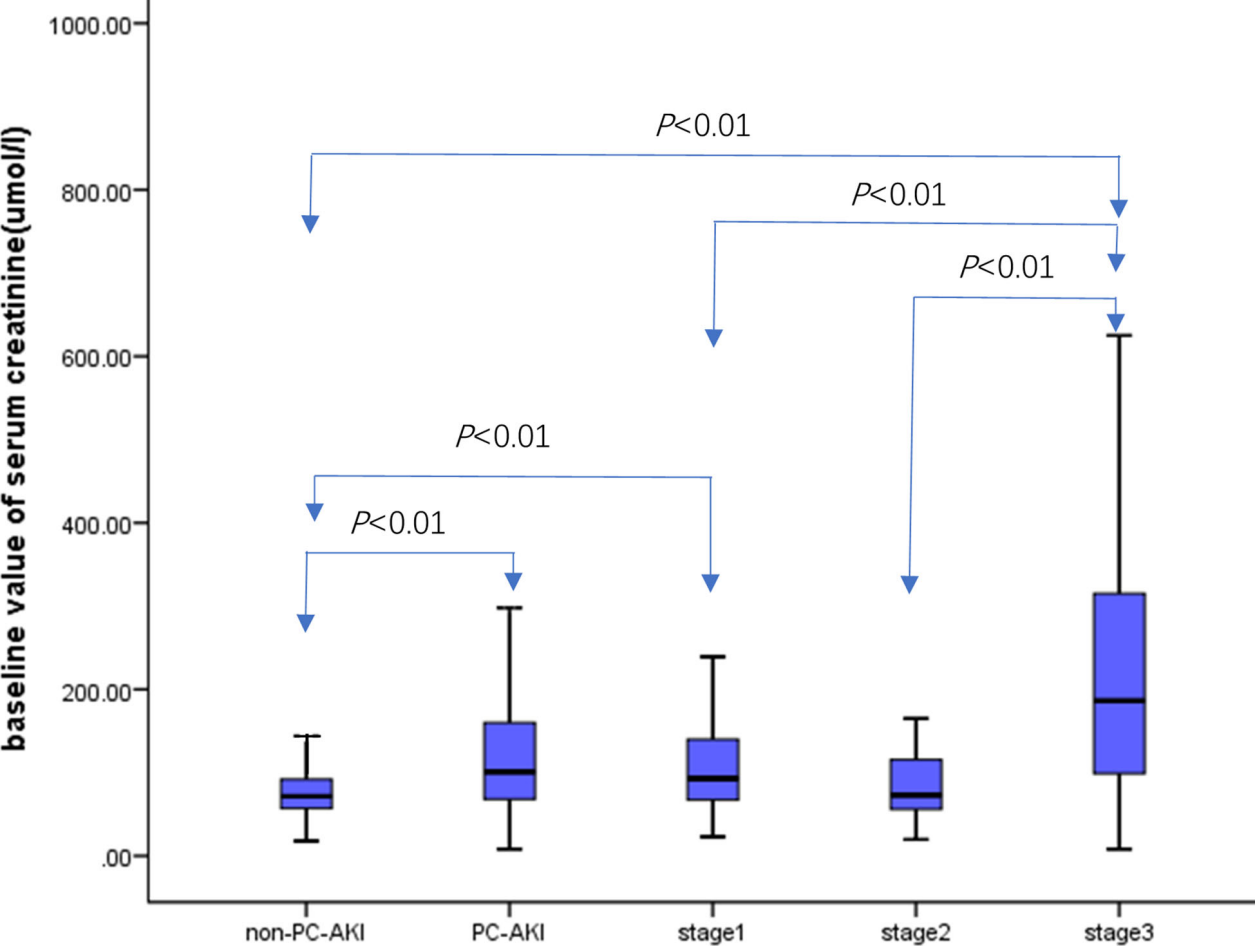
Comparison of baseline value of serum creatinine in different PC-AKI stages.

There were 5,545 cases in the non-PC-AKI group, and the baseline value of creatinine was 71.60 [57.10, 92.00] µmol/L. Meanwhile, there were 366 cases in the PC-AKI group, and the baseline value of creatinine was 101 [68.00, 160.45] µmol/L. The baseline creatinine level in the PC-AKI group was higher than that in the non-PC-AKI group (*P*<0.01). There were 258, 37, and 71 cases in stages 1–3, respectively. The baseline creatinine values were 93.00 [67.0, 140.25] µmol/L, 73.00 [56.00,1 16.05] µmol/L, and 186.30 [92.0, 315.30] µmol/L in stages 1–3, respectively. The baseline SCr level in stage 1 group was higher than that in the non-PC-AKI group (*P*< 0.01). Meanwhile, the baseline SCr level of stage 3 group was higher than that of the non-PC-AKI, stage 1, and stage 2 groups (*P*<0.01) (**Figure 4**).

### 3.7 Comparison of creatinine baseline values in patients with PC-AKI within 3 days and 4–7 days after using CM

A total of 366 cases of PC-AKI occurred within 3 days after using CM, with a baseline SCr value of 101 [68.00, 160.45] µmol/L. Meanwhile, 151 PC-AKI cases occurred within 4–7 days, with a baseline SCr value of 85 [60.00, 145.00] µmol/L. The difference in baseline SCr values between the two groups was statistically significant (*P*=0.022).

### 3.8 Risk factors of PC-AKI

Acoording to the results of multivariate analysis, hypertension, CKD, heart failure, peripheral vascular disease, metformin, diuretics, and CM dose were identified as risk factors for PC-AKI (**Table 4**).

**Table 4:** Risk factors of PC-AKI using binary logistic regression analysis.

Independent variables	*p-*value	Odds ratio	95% CI of OR^a^
age	0.801	0.999	0.990-1.008
sex	0.058	0.798	0.632-1.008
hypertension	0.000	1.895	1.479-2.429
chronic kidney disease	0.002	2.668	1.441-4.939
heart failure	0.003	1.477	1.137-1.919
peripheral vascular disease	0.022	0.588	0.373-0.927
metformin	0.010	0.628	0.440-0.897
diuretics	0.000	2.919	2.304-3.696
dose of contrast medium	0.047	1.292	1.003-1.665

a 95% CI of OR, 95% confidence interval of odds ratio; PCI-AKI, post-contrast acute kidney injury.

## 4 Discussion

With the development and advancement of imaging technology, iodine CM has been widely used in clinical practice. PC-AKI is the third leading cause of acute renal injury after renal hypoperfusion (42%) and postoperative renal injury (18%) (12) in hospitalized patients (1–4, 13, 14). However, the data showed that clinicians have paid insufficient attention to PC-AKI. In this study, CM was used in a total of 29,081 cases, but SCr levels were measured before and within 72 h after its application in only 6,418 cases (22.06%). The degree of concern for PC-AKI was relatively low, and measures should be taken to increase awareness and attention, and prevent the occurrence of PC-AKI.

Current literature reports that the incidence of PC-AKI ranges from 0% to 24%. The large difference in incidence may be closely related to differences in the diagnostic criteria (15–19). In this study, three SCr detection standards were used. The results showed that with an increase in the time required for SCr detection before and after CM application and an increase in the frequency of SCr detection, the incidence of PC-AKI increased significantly from 6.19% to 22.22%. To date, the baseline SCr level with respect to PC-AKI has not been clearly defined, which may lead to an increase in the diagnostic rate of PC-AKI. Therefore, SCr detection should be performed within 24 hours before the use of CM as the baseline value, and the frequency of SCr detection should be increased after the use of CM to improve the accuracy of PC-AKI diagnosis and reduce the misdiagnosis rate of PC-AKI.

The peak time for PC-AKI development was 48–72 h after the use of CM (10, 11, 20). However, in this study, SCr was detected daily from 1 to 7 days after CM administration, and the daily PC-AKI ratio was between 3.9% and 5.3%, which was similar (*P*>0.05). In other words, the proportion of daily PC-AKI within 1 week of the use of CM was similar to the incidence at 48–72 h, suggesting that the observation time should be further extended after the use of CM in clinical practice. SCr levels should be monitored daily for at least 1 week to avoid missed diagnosis of PC-AKI and occurrence of adverse events.

Hypertonic CM exhibits strong nephrotoxicity (21). With the development and replacement of hypotonic and isotonic CM, the occurrence of PC-AKI has effectively declined (22, 23). In this study, except for iodixanol, which was isotonic, the other six types of iodine CM were hypotonic. The incidence of PC-AKI in the seven types of CM was not significantly different (*P*>0.05). This suggests that there was no significant difference in the incidence of PC-AKI between hypotonic and isotonic iodine CMs, which is consistent with currently published guidelines (9) and the results of a meta-analysis in 2015 (24).

An increased CM dose is closely related to the occurrence of PC-AKI (9) and PC-AKI stages (25). CM dose max (Vmax) = 5 mL × body weight (kg)/basal SCr (mg/dL) (26). This study drew on similar models and Mehran scores (27) and defined a CM dose of ≥100 mL as an increase in the CM dose. The incidence of PC-AKI in the CM dose increase group (7.9%) was significantly higher than that in the non-increase group (5.7%) (P<0.01). Combining these results with published guidelines (28), the use of CM in the smallest necessary volume during the examination should be recommended. Previous studies have shown that the incidence of PC-AKI after DSA is high (29). In this study, the incidence of PC-AKI was 8.3% with DSA, which was higher than that with enhanced CT (5.8%), while the dose of CM used in DSA was higher than that in enhanced CT. This suggests that PC-AKI may be related to an increase in CM dose.

Patients with a high risk of developing PC-AKI include those with CKD and DM with impaired renal function (30). This study showed that the baseline SCr level in the PC-AKI group was higher than that in the non-PC-AKI group, that in the stage 1 group was higher than that in the non-PC-AKI group, and that in the stage 3 group was higher than that in the non-PC-AKI, stage 1, and stage 2 groups *(P*<0.01). This suggests that the higher the baseline SCr value, the greater the risk of PC-AKI (31) and the possibility of entering stage 3 PC-AKI. Advanced CKD with an eGFR less than 30 mL/min/1.73 m^2^ is a major cause of PC-AKI, resulting in a three-fold increase in the risk of PC-AKI (32). The eGFR is closely related to SCr levels.

Other associated risks, including hypertension, congestive heart failure, and volume depletion, can increase PC-AKI prevalence by up to 25% (30). Hypertension can lead to renal tissue ischemia, nephron loss, and a decrease in the number of effective nephrons and glomerular filtration rate, thus promoting PC-AKI (33). Congestive heart failure and diuretics can reduce the effective circulation volume and increase the release of vasoconstrictor hormones, which may lead to ischemia and hypoxia in the renal medulla and induce PC-AKI (34). Peripheral vascular disease is a risk factor for PC-AKI (35), which makes it more difficult to enter the blood vessels for examination or interventional therapy, and often requires a large amount of CMs. Metformin increases the risk of lactic acidosis (9). According to our data, metformin also increased the risk of PC-AKI. Therefore, it is better to be consistent with the guidelines (10) and to stop using metformin before using CMs.

The strengths of this study are as follows: 1) the relationship between the incidence of PC-AKI and different baseline SCr levels was explored; 2) the daily incidence of PC-AKI was counted to explore the time needed to observe whether PC-AKI occurred after the use of CM; and 3) the relationship between the baseline SCr value and PC-AKI and different stages of PC-AKI was analyzed. This study also has the following limitations: 1) retrospective studies are prone to selection bias; 2) the time span (10 years) of the selected samples was wide, for which the definition of PC-AKI changed over time, and related literature adopted a different definition.

## 5 Conclusions

The incidence of PC-AKI increased significantly with increasing time requirement and frequency of SCr detection. Therefore, based on Scheme 2, the frequency of SCr detection should be increased according to the characteristics of baseline SCr. Furthermore, an increase in the CM dose and SCr baseline value can increase the incidence rate of PC-AKI. Clinically, before using CMs, blood pressure and heart failure should be controlled, metformin and diuretics should be stopped, and CMs should be used at the minimum dose to avoid PC-AKI.

## Data availability statement

The raw data supporting the conclusions of this article will be made available by the authors, without undue reservation.

## Ethics statement

The studies involving human participants were reviewed and approved by Clinical Medical Ethics Review Committee of West China Hospital, Sichuan University. Written informed consent for participation was not required for this study in accordance with the national legislation and the institutional requirements.

## Author contributions

XR and XZ were responsible for study conception and design. JY, JW, and ZY were responsible for data and scheme management. ZY, JW, and JY performed the data cleaning and analysis. XR, XZ, JW, and JY interpreted data. JY, XR, JW, ZY, XZ, DC, YG and LZ drafted and revised the manuscript. All authors contributed to the article and approved the submitted version.
